# Characterizing and Exploring the Formation Mechanism of Salt Deposition by Reusing Advanced-softened, Silica-rich, Oilfield-produced Water (ASOW) in Superheated Steam Pipeline

**DOI:** 10.1038/srep17274

**Published:** 2015-11-26

**Authors:** Bin Dong, Ying Xu, Senmin Lin, Xiaohu Dai

**Affiliations:** 1 State Key Laboratory of Pollution Control and Resource Reuse, School of Environmental Science and Engineering, Tongji University, Shanghai, 200092, China; 2 Karamay Oilfield, PetroChina Ltd., Karamay, Xinjiang, 834000, China

## Abstract

To dispose of large volumes of oilfield-produced water, an environmentally friendly method that reuses advanced-softened, silica-rich, oilfield-produced water (ASOW) as feedwater was implemented via a 10-month pilot-scale test in oilfield. However, salt deposition detrimental to the efficiency and security of steam injection system was generated in superheated steam pipeline. To evaluate the method, the characteristics and formation mechanism of the deposition were explored. The silicon content and total hardness of the ASOW were 272.20 mg/L and 0.018 mg/L, respectively. Morphology and composition of the deposition were determined by scanning electron microscope**–**energy dispersive spectrometry (SEM-EDS), inductively coupled plasma**–**mass spectroscopy (ICP-MS), X-ray diffraction (XRD), laser Raman spectroscopy (LRS) and X-ray photoelectron spectroscopy (XPS). Na_2_Si_2_O_5_, Na_2_CO_3_ and trace silanes were identified in the deposition. In addition, the solubility of the deposition was about 99%, suggesting that it is very different from traditional scaling. The results of a simulation experiment and thermal analysis system (TGA and TG-FTIR) proved that Na_2_CO_3_ and Si(OH)_4_ (gas) are involved in the formation of Na_2_Si_2_O_5_, which is ascribed mainly to the temperature difference between the superheated steam and the pipe wall. These findings provide an important reference for improving the reuse of ASOW and reducing its deposition.

Large volumes of produced water are generated as a by-product during the exploitation of oil in most Chinese oilfields. The 18th well area of Fengcheng Oilfield Work Zone in the Karamay Oilfield, for example, produces 40,800 m^3^/d of water. However, the daily global generation of produced water is 250 million barrels, which is three times the amount of oil produced, and this ratio increases with the maturity of the oilfield^1^,^2^. Oilfield-produced water is a complex mixture of oil, water and dissolved and suspended solids^3^, which can be immensely detrimental to ecology and the environment. Therefore, the disposal of large volumes of oilfield-produced water is an urgent and stubborn problem. However, from the ecological, environmental and economic standpoints, the most effective disposal method would be to reuse it in the steam injection system of the oilfield^4^,^5^.

One of the key challenges for reusing the produced water as boiler feedwater in oilfields is deposition, especially insoluble silicate deposition in the steam injection system, which is detrimental to the thermal efficiency and security of the system^6^,^7^,^8^,^9^. Therefore, stringent industry standards have been implemented to control the silicon content of feedwater^10^,^11^,^12^. British Standards limit the maximum silicon content to 5 mg/L (calculated with SiO_2_) with a pressure range of 6.1–8.0 MPa in fire water tube boilers. To meet these stringent industry standards, many techniques are in common use to remove silica from water^13^,^14^. However, all these methods are restricted because they produce large quantities of silica sludge and concentrated solutions, which are prone to causing secondary pollution and increasing the financial cost of treatment. In China, desilication costs account for more than half of the total operating cost of oilfields^15^. In our previous work, we found softening to be more efficient than desilication in reducing the deposition in lab-scale tests, and the formation of SiO_2_ in the deposition was negligible even when the boiler was operated at a high silicate concentration^15^,^16^. Nevertheless, the steam injection system in oilfields is mainly composed of a convection section (finned tubes), a radiation section (radiation tubes) and a superheated steam pipeline. For a systematic study, we need to discuss each of the sections. According to the practical industrial production, the superheated steam pipeline is most liable to form insoluble silicate deposition. However, the effect of reusing advanced-softened silica-rich oilfield-produced water (ASOW) on superheated steam pipelines is unknown,

Silica can carry over into the steam in two ways^6^. It can be present in the steam as the result of general boiler water carry-over, which can lead to scaling in the finned and radiant tubes, or it can enter the steam in a volatile form. It is well known that saturated steam is prone to carrying silicate, and it has been conclusively established that saturated steam will carry silica as a volatile compound^17^,^18^. In addition, at a given pressure, dry saturated steam continues to be heated and the temperature increases above its saturation temperature, forming superheated steam^19^. The superheated steam can carry a lot of silica as a volatile compound from the saturated steam. Thus, the silica may be deposited on the internal surface of a superheated pipeline^6^. This insoluble deposition (silica) can impede the flow of superheated steam and be detrimental to the security of the pipeline. However, the composition of the deposition formed by ASOW in a superheated steam pipeline is unknown. Is it a simple superposition of insoluble silicate or a new substance? How does it form? Few studies have explored the characteristics and formation mechanism of this salt deposition.

In this study, advanced-softened silica-rich oilfield-produced water (ASOW) was reused as boiler feedwater in a 10-month pilot-scale test. The basic water quality parameters were monitored by IC and TOC analyser. Scanning electron microscope–energy dispersive spectrometry (SEM-EDS), inductively coupled plasma–mass spectrometry (ICP-MS), X-ray diffraction (XRD), laser Raman spectroscopy (LRS) and X-ray photoelectron spectroscopy (XPS) were applied to analyse the characteristics of the salt deposition. According to these data, the morphology and elemental and compound compositions of the salt deposition were explored. The solubility of the deposition was studied via solubility experiments. Moreover, the formation mechanism of the salt deposition was examined by a simulation experiment and thermal analysis system (TGA and TG-FTIR). While developing a clear understanding of the salt deposition formed by ASOW is obviously of great importance to reduce deposition in superheated steam pipelines, the findings can also be used to evaluate and improve the reuse of ASOW in steam injection systems. This study also offers a reference for energy conservation and emissions reduction.

## Results

### Feedwater Quality Results

The quality of the ASOW boiler feedwater was monitored, and the raw data are summarised in the Supplementary Information (Figure S4 and Tables S2, S3). The average values and the numerical range of the general feedwater quality parameters are summarised in Table S4, Supplementary Information. The average silicon content (calculated with SiO_2_) and total hardness (calculated with CaCO_3_) in the ASOW were 272.2 mg/L and 0.018 mg/L, respectively, while the average total concentration of metal ions (Ca, Mg, Al and Fe), which are prone to scaling, was only 0.021 mg/L. In addition, large amounts of bicarbonate radicals (442 mg/L) were found in the ASOW.

### Dissolution Characteristics of the Salt Deposition

The change in solubility of the salt deposition over time is summarised in the Supplementary Information (Figure S5). Table 1 provides the basic dissolution characteristics of the salt deposition. The salt deposition can be mostly dissolved but needs a long time, and the solution is strongly alkaline. In addition, most of the C elements exist in inorganic compounds due to the TIC/TC ratio (97.92%). Meanwhile, the TOC content of the deposition was only 0.14 mg/g (Table 1), indicating that the organic compounds were negligible.

### Elemental Composition and Compounds

**Figure 1:**
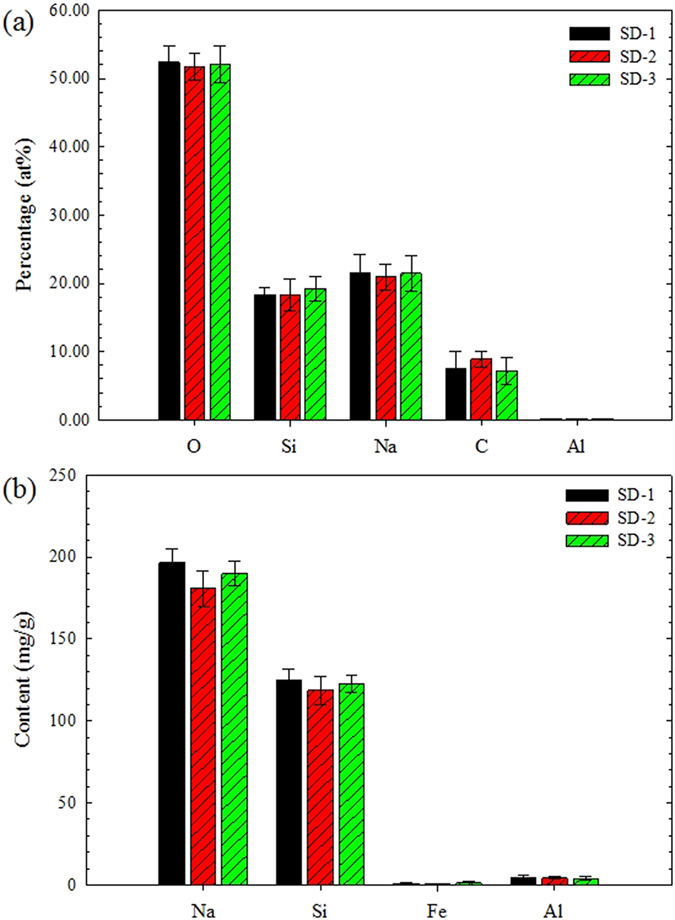
Elemental composition of samples SD-1, SD-2 and SD-3: (a) elemental percentage in powder samples by EDS analysis; (b) elemental content of digested samples by ICP-MS analysis.

SEM images and the raw EDS data for the powder samples (SD-1, SD-2 and SD-3) and ICP-MS data for the digested powder samples are summarised in the Supplementary Information (Figure S6 and Tables S5 and S6). The EDS and ICP-MS analysis indicated that the most frequently detected elements in the salt deposition were O, Si, Na, C and a small quantity of Al (Fig. 1 (a,b)). As shown in Fig. 1, there were no obvious differences in the elemental content of the SD-1, SD-2 and SD-3 samples. The mole percentages of the four main elements (O, Si, Na and C) in the salt depositions were about 52.0%, 18.6%, 21.3% and 7.9%, respectively. In addition, the Na content was about 190 mg/g and the molar ratio of Na/Si was about 1.6 (Fig. 1(b)).

**Figure 2:**
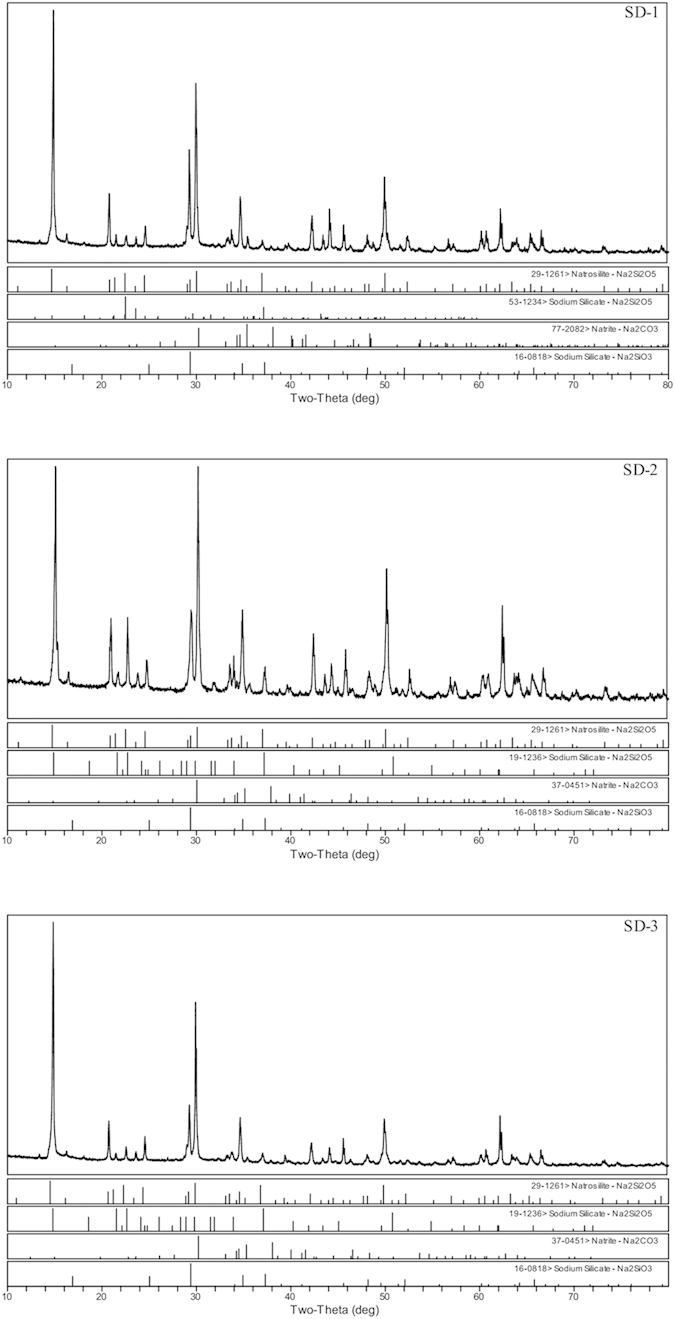
XRD patterns of the powder samples from SD-1, SD-2, and SD-3.

The XRD patterns of the three representative salt depositions are provided in Fig. 2, and the semi-quantitative results are summarised in the Supplementary Information (Table S7). Natrosilite (Na_2_Si_2_O_5_), sodium silicate (Na_2_Si_2_O_5_ and Na_2_SiO_3_) and sodium carbonate (Na_2_CO_3_) were the main crystalline minerals in the salt deposition. In addition, the degree of natrosilite (Na_2_Si_2_O_5_) matched most closely among the three samples (Fig. 2). However, the depositions from SD-1, SD-2 and SD-3 were consistent in their main compounds; thus any one of the three samples is representative.

**Figure 3:**
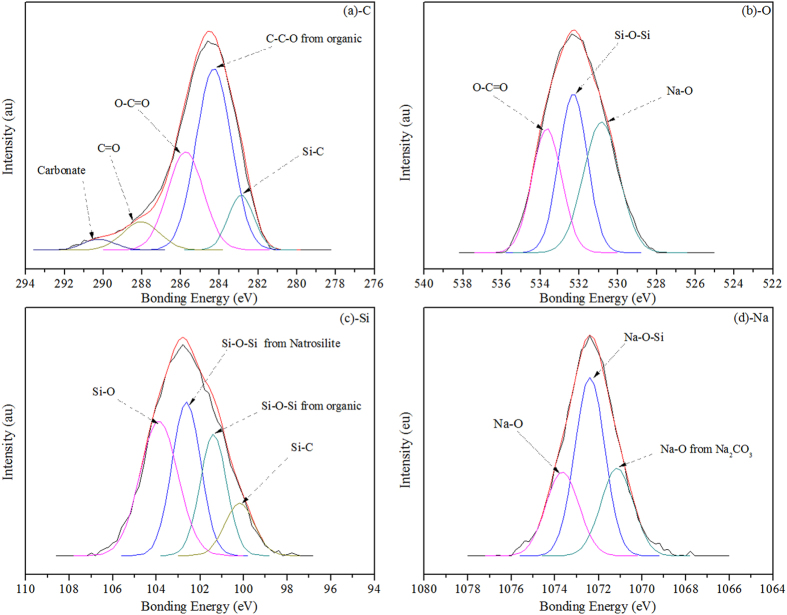
XPS spectra for C (a), O (b), Si (c) and Na (d) in the salt deposition powder sample.

The raw data for the XPS of the salt deposition powder sample are summarised in the Supplementary Information (Figure S7). XPS spectral analyses for C, O, Si and Na in the sample are provided in Fig. 3. The XPS results were analysed according to the Standard Reference Database and the appropriate literature^20^,^21^. The C 1s XPS region confirmed the assessment of a mixture of carbonate, silanes and some other organic compounds. The C 1s XPS region shown in Fig. 3(a) mainly indicates three different carbon-containing species. The most intense peak at 284.8 eV is attributed to the carbon of the anhydrides of aliphatic polymers. The peak at 285.6 eV is the O-C=O bond of polyacrylic acid. The third peak at 282.90 eV corresponds to the C-Si bond of silicon carbide. In addition, the peaks at 288.04 and 290.00 eV resulted from a carbonyl ligand and a carbonate, respectively.

Figure 3(b) shows the O 1s XPS spectrum, which can be fitted to three peaks with binding energies of 530.80 eV, 532.40 eV and 533.78 eV. The most intense peak at 532.40 eV is attributed to the Si-O-Si of sodium silicate. In addition, the peak resulting from the oxygen in polymethacrylic acid is found at a binding energy of 533.78 eV, and the binding energy peak at 530.80 eV is attributed to Na-O. These results indicate that the main chemical form of O in the deposition exists as sodium silicate, some organic compounds (silanes and polyacrylic acid) and carbonates.

In Fig. 3(c), the Si 2p spectrum consists of four peaks centred at 100.20 eV, 101.40 eV, 102.65 eV and 103.90 eV. These peaks can be attributed to silanes and disilicate. The most intense peak at 102.65 eV is ascribed to the Si-O-Si of the disilicate. The remaining three peaks at 100.20 eV, 101.40 eV and 103.90 eV are due to the Si-C bond of the silanes, the Si-O-Si bond of hexamethyldisiloxane and the Si-O bond of silica, respectively.

The Na 1s XPS spectrum of the SD-2 powder sample is shown in Fig. 3(d). The maximum photoelectron signal appears at 1072.70 eV, which is attributed to the Na-O-Si of the disilicate. In addition, the peaks at 1071.50 eV and 1073.40 eV are attributed to the Na-O bonds of Na_2_CO_3_ and NaFeO_2_, respectively.

### Structural Morphology of the Solid Deposition

**Figure 4:**
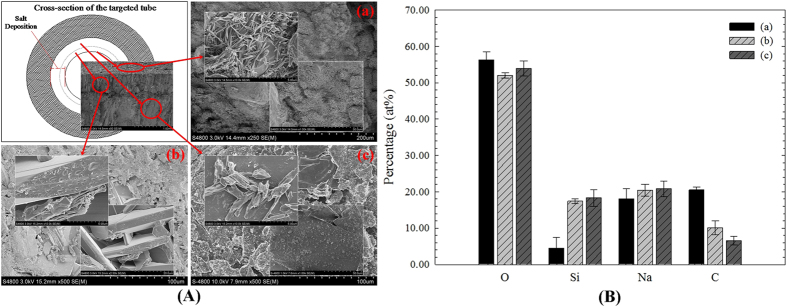
Structural morphology and main elemental composition of the solid sample: (A) layered structure; (B) corresponding elemental composition.

**Figure 5:**
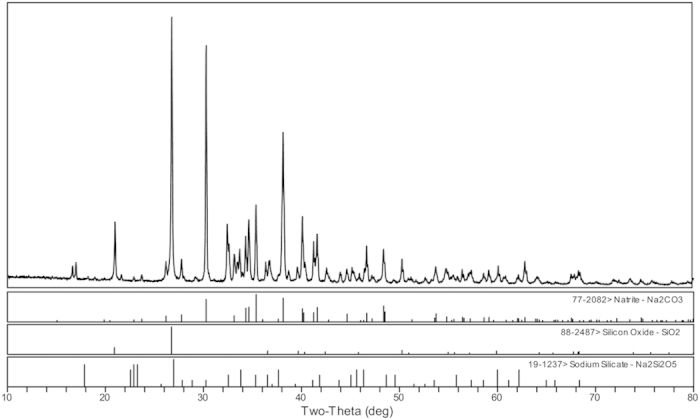
XRD pattern of the cluster crystals on the surface of the deposition.

The structural morphology of the solid sample was studied by SEM-EDS. Figure 4 provides the basic layered structure and corresponding elemental composition of the deposition. A distinct three-layered structure is presented in Fig. 4(A). Some cluster crystals appeared on the surface of the deposition (Fig. 4A (a)), whereas block-like crystals appeared in the middle (Fig. 4A (b)) and vitreous crystals formed on the internal surface of the pipeline (Fig. 4 A (c)). However, there was a big difference between (a) and (b) in the relative content of the Si and C elements. The XRD pattern of the cluster crystal is presented in Fig. 5. Clearly, the cluster crystal was sodium carbonate (Na_2_CO_3_).

### Simulation Experiments and Characterisation

**Figure 6:**
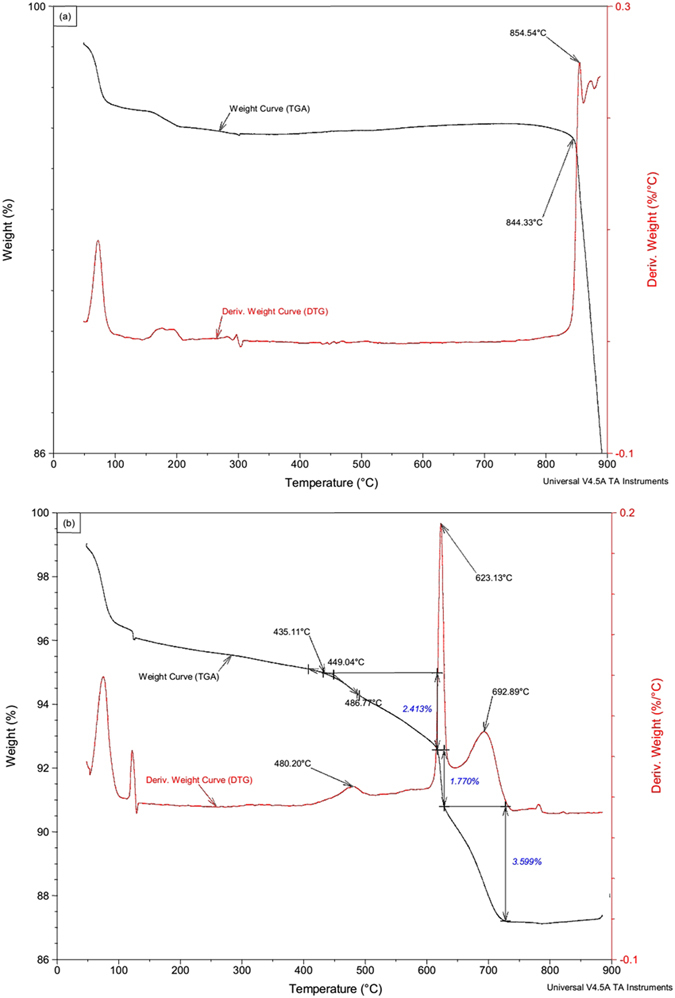
TGA and DTG curves of Na_2_CO_3_ (a) and a mixture of Na_2_CO_3_ and SiO_2_ (b).

The TGA curves and corresponding DTG curves of the Na_2_CO_3_ and the mixture (the molar ratio of Na/Si was about 1.6) are shown in Fig. 6. In addition, the CO_2_ produced in the mixture at different times was identified by TG-FTIR in the Supplementary Information (Figure S8). The Na_2_CO_3_ had an obvious weight loss peak at 855 ± 1 °C in the DTG curve (Fig. 6(a)), suggesting that this is the maximum decomposition temperature. In addition, the onset decomposition temperature of Na_2_CO_3_ was about 844 ± 1 °C. Figure 6(b) shows three obvious weight losses (2.4%, 1.77% and 3.6%) in the TGA curve, and three corresponding peaks (480 ± 1 °C, 623 ± 1 °C and 693 ± 1 °C) in the DTG curve at 300–800 °C, which are attributed to the maximum decomposition temperatures. However, the decomposition temperature of the Na_2_CO_3_ was decreased when mixed with SiO_2_, and decomposition occurred at 435 ± 1 °C for the first time. Moreover, the obvious peak at 2358 cm^−1^ attributed to the CO_2_ can be found at 435–680 °C (2609.9–4085.4 seconds) (Figure S8).

**Figure 7:**
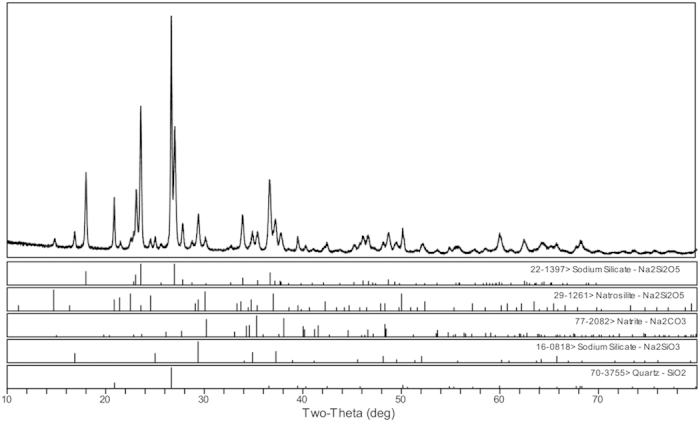
XRD pattern of the simulated product.

**Figure 8:**
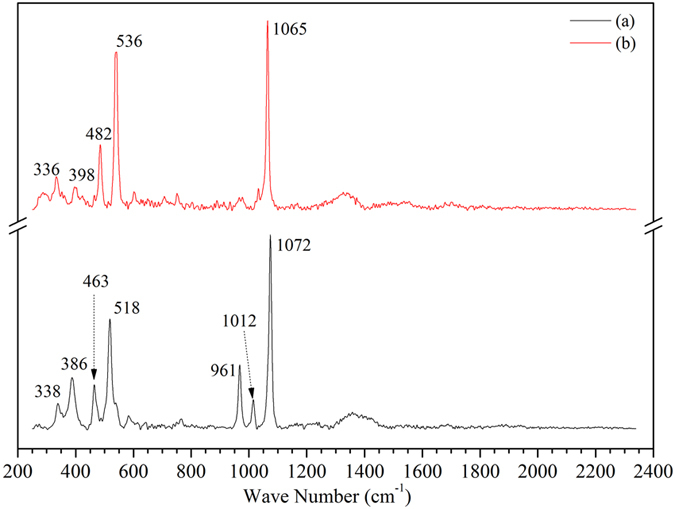
Raman spectra of the simulated product (a) and salt deposition (b).

The simulated products were characterised by XRD and LRS. As shown in Fig. 7, sodium silicate (Na_2_Si_2_O_5_), natrosilite (Na_2_Si_2_O_5_) and sodium carbonate (Na_2_CO_3_) were the main crystalline phases identified in the simulated products. In addition, the degree of matching was greatest for the sodium silicate (Na_2_Si_2_O_5_). The Raman spectra of the salt deposition and the simulated products are presented in Fig. 8 and show similar patterns, suggesting that the main compounds have similar structural units. The strong Raman bands at 1073 cm^−1^ (Fig. 8(a)) and 1065 cm^−1^ (Fig. 8(b)), which are usually associated with the stretching vibrations of the terminal nonbridging oxygens (ν(Si-O^−^)) of the Na_2_Si_2_O_5_ species, appear in the 1100–1050 cm^−1^ region^22^. Meanwhile, the bands at 463 cm^−1^, 518 cm^−1^, 482 cm^−1^ and 536 cm^−1^ are presented in the 700–400 cm^−1^ region, which is attributed to the stretch vibration of the Si-O-Si modes. In addition, the bands at 398 and 336 cm^−1^ are attributed to the Na-O vibration of the natrosilite (Na_2_Si_2_O_5_). In Fig. 8(a), the Na-O vibration of the sodium silicate (Na_2_Si_2_O_5_) is characterised at 386 and 338 cm^−1^, while the bands at 1012 cm^−1^ and 961 cm^−1^ can also be attributed to a small quantity of structural units in chains and dimmers^23^, respectively.

## Discussion

The amounts of amorphous silica, silicates and sodium bicarbonate found in the feedwater (ASOW) can be attributed to the removal of metal cations (such as Ca^2+^, Mg^2+^, Fe^2+^ and Fe^3+^) that are prone to scaling with silicates and carbonates. In the boiling water, sodium bicarbonate will be decomposed to Na_2_CO_3_, while the silica and silicates can be converted to Si(OH)_4_ (gas) in the saturated steam at 12.80 MPa and 329 °C, which are the pressure and temperature in the radiant section of the test boiler^17^,^24^,^25^,^26^. Thus, the silicates and silica can be carried as volatiles into the superheated steam. Meanwhile, the Na_2_CO_3_ can also be carried into the superheated steam by mechanical carry-over. Thus, Si(OH)_4_ (gas) and Na_2_CO_3_ can be present simultaneously in the superheated steam pipeline.

However, the salt deposition can be cleaned by water based on the solubility (Table 1), suggesting that the deposition is very different from traditional scaling. This is a positive result for the reuse of ASOW as feedwater in steam injection systems. Based on Table 1 and the surface analyses (Fig. 3), trace organic compounds (silanes and polyacrylic acid) are found on the surface of the inorganic crystal particles. However, according to the results of the EDS, XRD and XPS, the main compounds in the salt deposition are inorganic (Na_2_Si_2_O_5_ and Na_2_CO_3_), mostly the new compound (Na_2_Si_2_O_5_), suggesting that this is not simply the stacking of salts, but that some kind of reaction among the compounds has occurred. How does the Na_2_Si_2_O_5_ form? According to the results of the elemental and compound composition, it can be assumed that Na_2_CO_3_ reacts with SiO_2_ to form Na_2_Si_2_O_5_.

As is well known, SiO_2_ is stable below 870 °C^24^, and Na_2_CO_3_ does not decompose below 844 °C based on Fig. 6(a). However, there is an obvious weight loss at 435–680 °C in the TGA curve (Fig. 6(b)), and the CO_2_ produced at 435–680 °C was identified by TG-FTIR (Figure S8). These results indicate that the Na_2_CO_3_ started to decompose at 435 °C in the Na_2_CO_3_ and SiO_2_ mixture (the molar ratio of Na/Si is about 1.6) as shown in Eq. (1). Thus, at 486 °C, it can be inferred that Na_2_O can react with SiO_2_ to form Na_2_Si_2_O_5_, which is confirmed by the results of the simulation experiment (Figs 7 and 8). In addition, there was a slight difference in the form of Na_2_Si_2_O_5_ between the salt deposition and the simulated experiment (Figs 2,7 and 8), indicating that the form in which it exists is mostly influenced by the pressure.


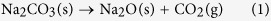


**Figure 9:**
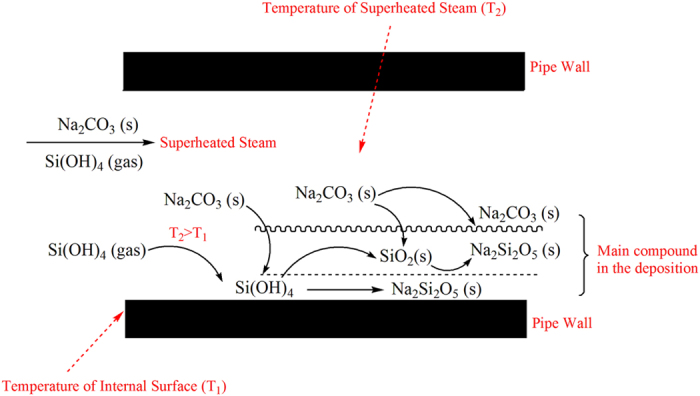
Schematic diagram of the main compound forming process of the deposition.

According to the results in Figs 4 and 5, Na_2_CO_3_ is the main compound on the surface of the deposition. Thus, it can be concluded that Na_2_Si_2_O_5_ of different crystal morphologies is the main compound in the other locations (Fig. 4(A (b–c))). Meanwhile, the vitreous microstructure and elemental composition (the molar ratio of Na, Si and O was about 1:1:2.5) also confirm this. According to the foregoing analysis, the formation process of Na_2_Si_2_O_5_ can be described. As shown in Fig. 9, due tothe temperature difference between T_2_ and T_1_, gaseous Si(OH)_4_ with phase transformation adheres to the internal surface of the pipeline. Then, it is dewatered and transformed into SiO_2_ when the boiler is operating. Meanwhile, Na_2_CO_3_ brought by mechanical carry-over is mixed with the SiO_2_ by the air currents. At 486 °C and 12.65 MPa, a different form of Na_2_Si_2_O_5_ is formed.

An environmentally friendly method for disposing of large volumes of oilfield-produced water was implemented via a pilot-scale test in an oilfield, which did not generate large quantities of silica sludge and concentrated solutions. The effect of the ASOW on the superheated steam pipeline was positive based on the characteristics of the salt deposition. It is soluble and can be removed by water, which is very different from traditional scaling, suggesting that reusing the ASOW as feedwater can offer an important reference for disposing of the large volumes of oilfield-produced water. It is also important for the reduction of environmental pollution and the preservation of water resources. In addition, because of the formation mechanism of the salt deposition, reducing the temperature differential between the superheated steam and the pipe wall can decrease the opportunity for Si(OH)_4_ (gas) to be converted to SiO_2_, and thus greatly control the amount of salt deposition.

## Methods

### Experimental System

A pilot-scale test was implemented for 10 months. The disposal process flow diagram for oilfield-produced water, photographs and basic parameters of the superheated steam boiler for the pilot-scale test are summarised in the Supplementary Information (Figures S1 and S2 and Table S1). A new superheated steam tube, pre-processed by impeller blasting, was connected to the superheated steam pipeline as the target tube prior to the test. The temperature and pressure of the target tube were set at 486 °C and 12.65 MPa, respectively, in accordance with the industrial production parameters of oilfields.

### Feedwater Quality

The total hardness of the feedwater (ASOW) was monitored daily over the 10-month period. Other important water quality parameters were measured aperiodically. Total hardness was determined by HACH DR2800 and the silicon content was measured by molybdenum blue^27^. The oil content, total dissolved solids (TDS) and total alkalinity (TA) were determined by the national standard method^12^,^28^ and Standard Method 2540^27^. The inorganic elements (e.g. Na, Ca, Mg, Al and Fe) in the feedwater were quantitatively analysed via inductively coupled plasma-mass spectroscopy (Agilent 7700 ICP/MS) in accordance with Standard Method 3125^27^. The other water quality parameters were determined using an IC (THermo scientific ICS2100), TOC analysis (SHIMADZU TOC-L CPH/CPN), portable conductivity meter (EUTECH ECTestr11+), and pH meter (METTLER TOLEDO-S20). All the measures were duplicated and the results show the mean of the duplicated samples with their standard deviation.

### Sample Collection and Pre-processing

The targeted tube was cut after the test. For representativeness and accuracy of sampling, samples of the salt deposition were taken from three positions, namely, SD-1, SD-2 and SD-3, on the internal surface of the tube, as shown in Supplementary Information (Figure S3). In addition, the SD-2 sample was studied in greater detail because it had more deposition than the other two sections and the temperature and pressure detectors were there. Each of the solid samples (SD-1, SD-2 and SD-3) was divided into two parts. One part of the solid sample was reserved and the other part ground into a powder that could pass through the griddle at the 0.075 mm pore rating by agate mortar. Parts of the powder samples (weighing about 0.2000 ± 0.0005 g) were digested with microwave-assisted hydrofluoric acid^29^ in accordance with Standard Method 3030^27^, after which the digestive solution was transferred to a 100-mL volumetric flask. This analysis was repeated for all samples (SD-1, SD-2 and SD-3) to avoid sampling uncertainty. In addition, samples of feedwater were collected aperiodically and transported under refrigeration to the laboratory. All liquid samples were stored at 4 °C before utilisation.

### Characteristics of the Salt Deposition Solution

An appropriate SD-2 solid sample and qualitative filter papers were vacuum-dried at 105 °C for 2 h, cooled to ambient temperature and weighed respectively (m_1_ and m_2_). The SD-2 solid sample (m_1_ = 4.0250 g) was weighed into a 500-mL beaker, and 200 mL of ultra-pure water was added directly to the beaker and left for 48 h. Then, the solution was filtered by the weighed qualitative filter paper. The filtrate was collected and analysed by TOC analysis and a pH meter. In addition, the qualitative filter paper and retentate were vacuum-dried at 105 °C for 2 h, cooled to ambient temperature and weighed (m_3_). Thus, the solubility was calculated by the change in weight before and after filtration, as shown in the following formula:


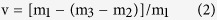


where m_1_ is the weight of the SD-2 solid sample, m_2_ is the weight of the qualitative filter paper and m_3_ is the combined weight of the qualitative filter paper and the retentate.

### Simulation Experiment

The appropriate anhydrous sodium carbonate (Na_2_CO_3_) and silicon dioxide (SiO_2_) powders were vacuum-dried at 105 °C for 2 h, and then cooled to ambient temperature. Then, 5.3000 g of Na_2_CO_3_ and 3.7500 g of SiO_2_ were weighed into a 500-mL beaker, and 100 mL of ultra-pure water was added. The solution was stirred with a magnetic stirrer at room temperature for 1 day. After drying the solution, the resultant solid (RS) was pulverised. Part of the RS powder was used to explore the reaction process of Na_2_CO_3_ and SiO_2_ by the thermal analysis system (TGA). The rest of the RS powder was heated at 486 °C for 168 h in an air atmosphere to simulate the formation of Na_2_Si_2_O_5_.

### Analytical Methods and Parameters

The elemental composition of the salt deposition (SD-1, SD-2 and SD-3) was determined using the SEM-EDS (Hitachi S-4800) technique and ICP-MS. XRD (Bruker D8 Advance) measurements were performed on the three powder samples (SD-1, SD-2 and SD-3), the surface of the SD-2 solid sample, and the RS powder sample to identify their predominant mineralogical phases. The range of the 2θ values was 10–80° with a 0.05° step size. The scanning speed was 2°per minute. The XRD patterns were identified using Jade^+^ (Version 6.5) and a semi-quantitative analysis of the compounds was taken using the method of reference intensity (RIR)^30^. Morphological analysis was performed on the solid samples. The SEM data were acquired in two modes. The first mode allowed the entire morphology of all samples (SD-1, SD-2 and SD-3) to be examined at 2000× magnification. The second mode allowed the stratification analysis of SD-2 on the tube while EDS data were also collected.

To research the chemical states of the main elements in the salt deposition, XPS was performed on the SD-2 powder samples. The XPS experiments were carried out on an RBD upgraded PHI-5000C ESCA system (Perkin Elmer) using Mg Kα radiation (hν = 1253.6 eV) or Al Kα radiation (hν = 1486.6 eV). The whole spectra (0~1100 (1200) eV) and narrow spectra of all of the elements were recorded at high resolution using an RBD 147 interface (RBD Enterprises, USA) and AugerScan 3.21 software. The binding energies were calibrated using containment carbon (C1s = 284.6 eV). The data analysis was carried out using RBD AugerScan 3.21 software provided by RBD Enterprises.

A laser Raman spectroscope (Pro-TT-EZRaman-B2) was used to identify and explore the molecular structural properties of the main compounds in SD-2 and the RS powder samples in the 250–2350 cm^−1^ region. The excitation line at 785 nm was taken with a diode laser. Its scan number is 80, and the resolution is 1.3 cm^−1^. The Raman spectra were analysed by Spectral Interpretation^31^ and Group Frequencies^32^,^33^. In addition, SDT-Q600 (TGA-DTG) and STA-6000 (TG-FTIR) apparatus were used to explore the weight loss of the mixture of Na_2_CO_3_ and SiO_2_. The thermal analysis was performed with a heating rate of 10 °C/min from 50 to 900 °C under a flow of dry air with alumina as the reference. The results were analysed using TA Universal Analysis software.

## Additional Information

**How to cite this article**: Dong, B. *et al*. Characterizing and Exploring the Formation Mechanism of Salt Deposition by Reusing Advanced-softened, Silica-rich, Oilfield-produced Water (ASOW) in Superheated Steam Pipeline. *Sci. Rep*. **5**, 17274; doi: 10.1038/srep17274 (2015).

## Supplementary Material

Supplementary Information

## Figures and Tables

**Table 1:** Basic characteristics of the salt deposition solution.

Indexes	Average Values	Average Deviation
Sample quality (g)	4.0250	0.0003
Volume of ultra-pure water (mL)	200	1
TOC (mg/L)	2.742	0.008
TC (mg/L)	238.6	4.3
pH (25°C)	11.42	0.16
Solubility	99%	0.004
Dissolution Time (h)	48	/
