# Supplementary material for: Characterizing and Exploring the Formation Mechanism of Salt Deposition by Reusing Advanced-softened, Silica-rich, Oilfield-produced Water (ASOW) in Superheated Steam Pipeline
Source: Sci Rep. 2015 Nov 26;5:17274. doi: 10.1038/srep17274 (PMC4660299; doi:10.1038/srep17274)
Supplement: Supplementary Information [file srep17274-s1.doc]

**Supplementary Information**

Characterizing and Exploring the Formation Mechanism of Salt Deposition by Reusing Advanced-softened, Silica-rich, Oilfield-produced Water (ASOW) in Superheated Steam Pipeline

**Bin Dong**^1,+^**,** **Ying Xu**^1,+,*^**,** **Senmin Lin**^2^**, Xiaohu Dai**^1,*^

^1^ State Key Laboratory of Pollution Control and Resource Reuse, School of Environmental Science and Engineering, Tongji University, Shanghai, 200092, China

^2^ Karamay Oilfield, PetroChina Ltd., Karamay, Xinjiang, 834000, China

* Co-corresponding authors: Ying Xu and Xiaohu Dai, E-mail address: [assassinxu@sina.cn](mailto:assassinxu@sina.cn) ; Tel.:+86 21 65981794; fax: +86 21 65983602.

^+^ Ying Xu and Bin Dong contributed to the work equally and should be regarded as co-first authors.


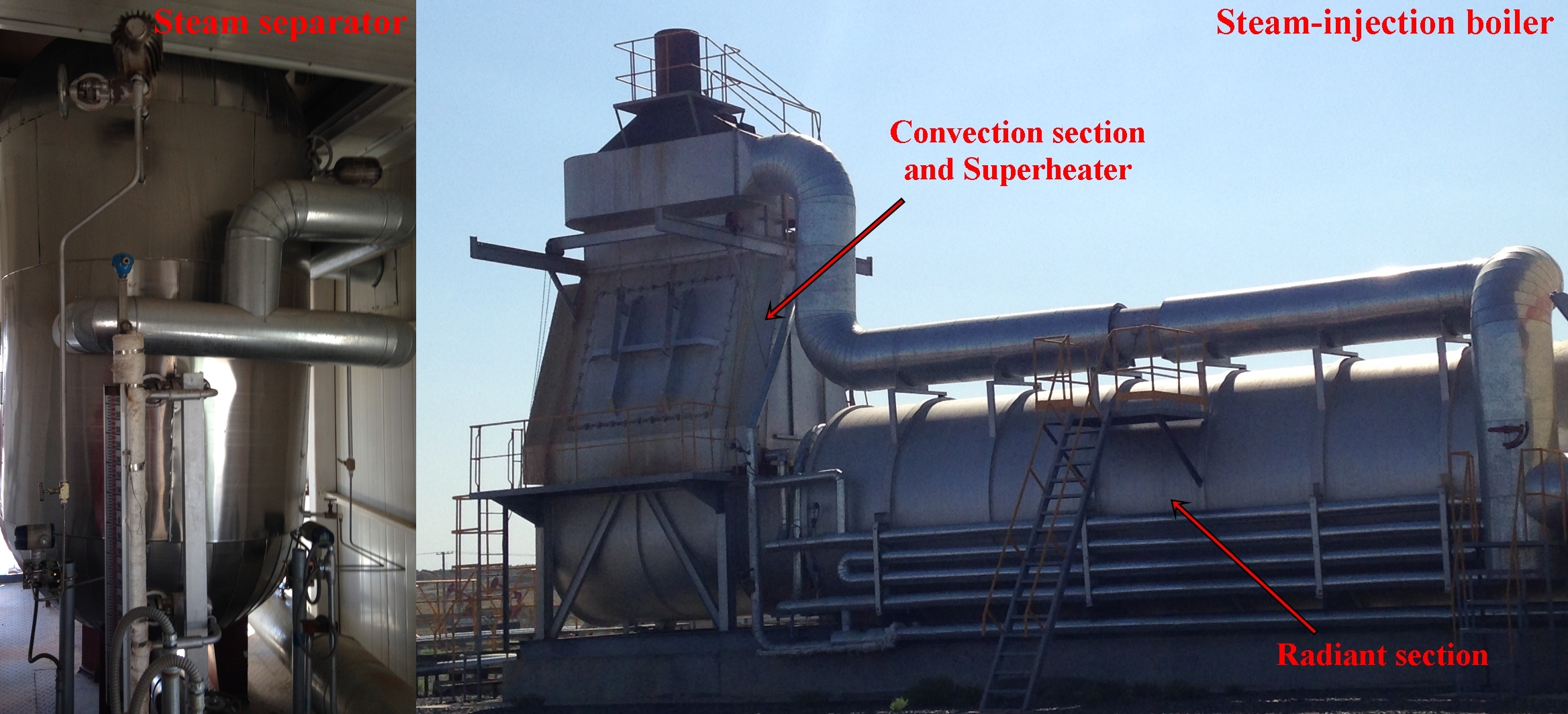


**Figure S1.** Photographs of the steam injection system (Steam separator and Steam-injection boiler)

**Table S1.** Basic parameters of the superheated steam boiler in pilot-scale test

| **Indexes** | **Values** |
| --- | --- |
| Model Number | YZG-14/360-G |
| Evaporation Capacity (t/h) | 20 |
| Thermal Efficiency | 86% |
| Rated Working Pressure (Mpa) | 14 |
| Dryness Fraction | 100% |
| Superheated Tube Material | 15CrMo alloy steel |
| Internal Diameter of Superheated Tube (mm) | 66 |
| Thickness of Superheated Tube (mm) | 12 |
| Average Superheated Steam Flow (t/h) | 12.15 |
| Pressure of Targeted Superheated Steam (Mpa) | 12.65 |
| Temperature of Targeted Superheated Pipeline (℃) | 486 |

**Figure S2.** Disposal process flow diagram of oilfield produced water in the pilot-scale test.

**Figure S3.** Samples of salt depositions were taken from three different positions named SD-1, SD-2, and SD-3 on the internal surface of the targeted tube

(a)

(b)

**Figure S4.** Total hardness of the feedwater was monitored for 10 months in the pilot-scale test. (a) 2013/6/1-2013/10/31; (b) 2013/11/1-2014/4/1

**Table S2.** The raw data of inorganic elements in feedwater (mg/L)

|  | Ca | Mg | Fe | Al | Na |
| --- | --- | --- | --- | --- | --- |
| 1 | 0.008 | 0.005 | 0.009 | 0.001 | 1481 |
| 2 | 0.005 | 0.008 | 0.004 | 0.003 | 1370 |
| 3 | 0.009 | 0.004 | 0.005 | 0.002 | 1311 |
| 4 | 0.007 | 0.005 | 0.003 | 0.009 | 1366 |
| 5 | 0.015 | 0.004 | 0.006 | 0.005 | 1501 |
| 6 | 0.009 | 0.007 | 0.003 | 0.001 | 1374 |
| 7 | 0.005 | 0.004 | 0.003 | 0.004 | 1371 |
| 8 | 0.007 | 0.007 | 0.005 | 0.003 | 1300 |
| 9 | 0.005 | 0.005 | 0.004 | 0.009 | 1412 |
| 10 | 0.013 | 0.010 | 0.003 | 0.001 | 1572 |
| 11 | 0.014 | 0.007 | 0.003 | 0.009 | 1678 |
| 12 | 0.009 | 0.004 | 0.004 | 0.003 | 1580 |
| 13 | 0.012 | 0.004 | 0.003 | 0.004 | 1403 |
| 14 | 0.013 | 0.008 | 0.005 | 0.009 | 1354 |
| 15 | 0.014 | 0.004 | 0.009 | 0.003 | 1308 |

**Table S3.** Parts of the water quality parameters in feedwater (units of content, mg/L, 25℃)

|  | **Silicon content (SiO_2_)** | **Oil content** | **Total Alkalinity (CaCO_3_)** | **Sulfate content** | **Bicarbonate content** | **Cl^-^** | **Conductivity (ms/cm)** | **pH** | **TDS** | **DO** |
| --- | --- | --- | --- | --- | --- | --- | --- | --- | --- | --- |
| 1 | 279 | 2.31 | 724 | 23.1 | 452 | 1067 | 5.52 | 7.22 | 3624 | 0.10 |
| 2 | 283 | 3.00 | 620 | 25.5 | 431 | 1081 | 5.98 | 7.81 | 4620 | 0.21 |
| 3 | 256 | 2.12 | 800 | 24.2 | 448 | 1109 | 5.00 | 8.12 | 4270 | 0.60 |
| 4 | 221 | 0.84 | 589 | 16.0 | 454 | 1000 | 5.89 | 8.09 | 3690 | 0.36 |
| 5 | 276 | 2.57 | 708 | 23.3 | 426 | 1265 | 6.00 | 9.00 | 4048 | 0.80 |
| 6 | 290 | 1.00 | 688 | 25.6 | 451 | 1289 | 5.77 | 8.17 | 3680 | 0.23 |
| 7 | 297 | 1.27 | 702 | 22.5 | 439 | 1267 | 5.78 | 7.71 | 5000 | 0.76 |
| 8 | 252 | 0.51 | 713 | 25.1 | 460 | 1043 | 5.90 | 8.10 | 3713 | 0.14 |
| 9 | 210 | 0.78 | 770 | 26.0 | 457 | 1276 | 6.00 | 7.40 | 4160 | 0.26 |
| 10 | 259 | 1.12 | 800 | 19.2 | 418 | 1189 | 5.96 | 8.23 | 4078 | 0.13 |
| 11 | 298 | 0.74 | 523 | 22.1 | 445 | 1283 | 5.91 | 8.11 | 3000 | 0.26 |
| 12 | 300 | 1.34 | 785 | 19.3 | 456 | 1300 | 5.86 | 7.50 | 4385 | 0.17 |
| 13 | 290 | 1.00 | 623 | 22.9 | 438 | 1274 | 6.00 | 7.42 | 3823 | 0.50 |
| 14 | 272 | 2.17 | 711 | 25.6 | 400 | 1288 | 5.97 | 8.07 | 3071 | 0.12 |
| 15 | 300 | 0.21 | 745 | 20.3 | 458 | 1269 | 5.90 | 7.16 | 3645 | 0.64 |

**Table S4.** The average values and the numerical range of general feedwater (ASOW) quality parameters in the pilot-scale test

| **Water Quality Indexes** | **Average Values** | **Standard Deviation** | **Numerical Range** |
| --- | --- | --- | --- |
| Silicon content（mg/L,SiO_2_） | 272.2 | ± 27.9 | 210~300 |
| Total Hardness（mg/L,CaCO_3_） | 0.018 | ± 0.009 | 0.007~0.030 |
| Oil content（mg/L） | 1.40 | ± 0.83 | 1.00~3.00 |
| Total Alkalinity（mg/L,CaCO_3_） | 700 | ± 80 | 500~800 |
| Conductivity（ms/cm,25℃） | 5.83 | ± 0.26 | 5.00~6.00 |
| Bicarbonate content（mg/L） | 442 | ± 17 | 400~460 |
| Cl^-^（mg/L） | 1200 | ± 107 | 1000~1300 |
| pH（25℃） | 7.87 | ± 0.48 | 7.50~9.00 |
| TDS（mg/L,25℃） | 3920 | ± 533 | 3000~5000 |
| DO（mg/L,25℃） | 0.35 | ± 0.24 | 0.10~0.80 |
| Ca（mg/L） | 0.010 | ± 0.003 | 0.005~0.015 |
| Mg（mg/L） | 0.006 | ± 0.002 | 0.004~0.010 |
| Al（mg/L） | 0.004 | ± 0.003 | 0.001~0.009 |
| Fe（mg/L） | 0.005 | ± 0.002 | 0.003~0.009 |
| Na（mg/L） | 1425 | ± 113 | 1300~1800 |

**Figure S5.** Changes of the solubility of salt deposition with time


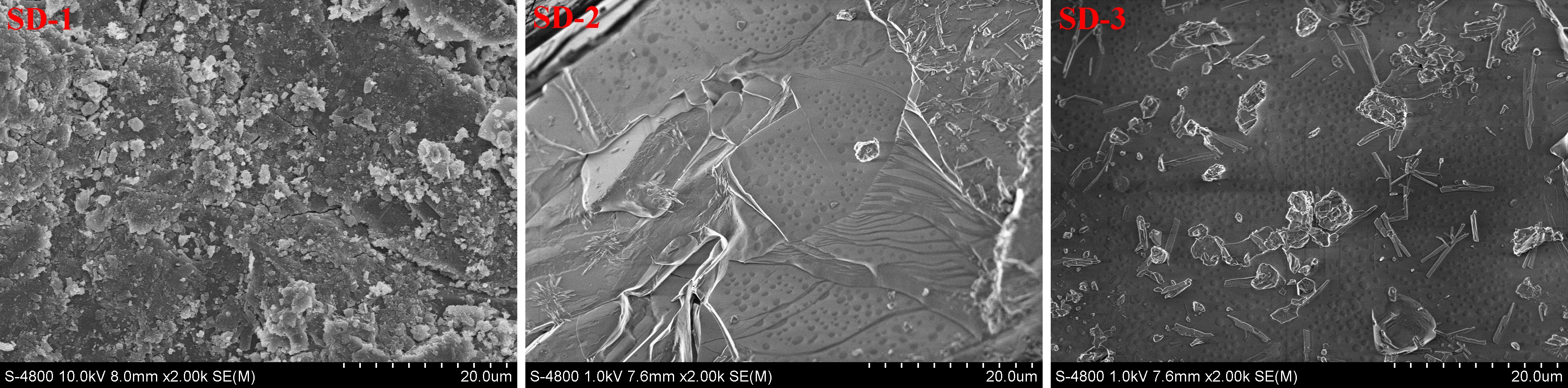


**Figure S6.** SEM images of powder samples from the SD-1, SD-2, and SD-3

**Table S5.** Raw EDS data of the samples from SD-1, SD-2, and SD-3

| Location of Tube | Times | Percentages of Elements (at%) | | | | |
| --- | --- | --- | --- | --- | --- | --- |
|  |  | **O** | **Si** | **Na** | **C** | **Al** |
| SD-1 | 1 | 53.10 | 18.38 | 20.50 | 7.85 | 0.17 |
|  | 2 | 52.02 | 19.16 | 21.25 | 7.45 | 0.12 |
|  | 3 | 51.16 | 19.07 | 22.07 | 7.51 | 0.19 |
| SD-2 | 1 | 51.79 | 19.26 | 20.01 | 8.78 | 0.16 |
|  | 2 | 50.43 | 17.91 | 22.35 | 9.21 | 0.10 |
|  | 3 | 52.56 | 18.23 | 20.42 | 8.67 | 0.12 |
| SD-3 | 1 | 52.75 | 18.28 | 21.62 | 7.23 | 0.12 |
|  | 2 | 50.96 | 20.41 | 21.17 | 7.33 | 0.13 |
|  | 3 | 52.01 | 19.62 | 20.97 | 7.19 | 0.21 |

**Table S6.** Raw data of the ICP-MS for digested samples from the SD-1, SD-2, and SD-3

| Location of Tube | | Percentages of Elements (mg/g) | | | |
| --- | --- | --- | --- | --- | --- |
|  |  | **Na** | **Si** | **Fe** | **Al** |
| SD-1 | 1 | 197.6 | 125.8 | 1.03 | 4.72 |
|  | 2 | 195.8 | 123.9 | 1.08 | 4.31 |
|  | 3 | 196.3 | 124.3 | 1.05 | 4.46 |
| SD-2 | 1 | 181.8 | 119.3 | 0.91 | 4.02 |
|  | 2 | 179.3 | 118.4 | 0.87 | 4.17 |
|  | 3 | 180.9 | 118.7 | 0.92 | 4.64 |
| SD-3 | 1 | 191.2 | 123.8 | 1.06 | 3.78 |
|  | 2 | 189.7 | 121.9 | 1.04 | 3.91 |
|  | 3 | 190.1 | 123.2 | 1.09 | 3.69 |

**Table S7.** Semi-quantitative results of XRD for the compound in the deposition

| **Crystalline phases** | **Percentages of Compounds (wt %)** | | |
| --- | --- | --- | --- |
|  | SD-1 | SD-2 | SD-3 |
| Natrosilite (Na_2_Si_2_O_5_) | 58 | 63 | 60 |
| Sodium Silicate (Na_2_Si_2_O_5_) | 19 | 18 | 23 |
| Sodium Silicate (Na_2_SiO_3_) | 10 | 10 | 9 |
| Na_2_CO_3_ | 7 | 6 | 6 |

**Figure S7.** Raw data of the XPS for SD-2 powder sample

(a)

(b)

(c)

**Figure S8.** TG-FTIR analysis for the mixture of Na_2_CO_3_ and SiO_2_ (the molar ratio of Na/Si is about 1.6): (a) FTIR spectrums of gases from the mixture at 435℃ (2609.94 seconds), 486℃ (2915.64 seconds), and 680℃ (4085.43 seconds); (b) Gases profile with different times; (c) Three-dimension profile of the gases from the mixture.
